# Intramedullary Abscess by* Staphylococcus aureus* Presenting as Cauda Equina Syndrome to the Emergency Department

**DOI:** 10.1155/2016/9546827

**Published:** 2016-05-16

**Authors:** Dimitrios Damaskos, Helene Jumeau, François-Xavier Lens, Philippe Lechien

**Affiliations:** ^1^Surgical Emergency Unit, John Radcliffe Hospital, Oxford University Hospitals NHS Foundation Trust, Oxford OX3 9DU, UK; ^2^Emergency Department, Hospital of Jolimont, rue Ferrer 159, Haine-Saint-Paul, 7100 La Louvière, Belgium

## Abstract

Cauda equina syndrome (CES) is a rare entity presenting with low back pain, unilateral or bilateral sciatica, motor weakness of lower extremities, sensory disturbance in the perineal area, and urinary and/or faecal incontinence. Those symptoms are secondary to compression of the cauda equina. If not recognized, CES can lead to irreversible disabilities. We report the case of a 77-year-old lady who presented to the emergency department with a ten-day history of back pain as well as urinary incontinence.

## 1. Introduction

Cauda equina syndrome (CES) is a rare syndrome presenting with low back pain, unilateral or bilateral sciatica, motor weakness of lower extremities, sensory disturbance in the perineal area, and urinary and/or faecal incontinence, resulting from compression of the cauda equina. It is one of the few spinal surgical emergencies and therefore is extremely important to recognize.

Causes of this syndrome are central disc prolapse; tumours (primary or metastatic); trauma (iatrogenic lumbar punctures, blunt trauma from fractures, severe disc herniation, spinal anaesthesia involving trauma from catheters and high local anaesthetic concentrations around the cauda equina, and penetrating trauma); lumbar spinal stenosis (osteoarthritis or spondylolisthesis); and inflammatory conditions (Paget disease, neurosarcoidosis, chronic inflammatory demyelinating polyneuropathy, ankylosing, spondylitis, tuberculosis, and intradural abscess). Spinal intradural abscess is a really rare condition, with a few cases described [1, 2].

We report the case of a 77-year-old lady who presented to the emergency department with a ten-day history of back pain as well as urinary incontinence as a result of an intramedullary abscess, causing cauda equina syndrome.

## 2. Case Report

A 77-year-old female patient presented to the emergency department, referred by her GP, with a ten-day history of back pain associated with intense radicular pain irradiating mainly to the right calf. Additionally, the patient complained of urinary incontinence during the last 15 days. There was no complaint of faecal incontinence. No episodes of fever or chills were reported.

The patient's medical history was remarkable for diabetes type II, hypertension, compensated alcoholic cirrhosis, a right hemicolectomy for adenocarcinoma of the colon, small bowel obstruction, and recurrent urinary tract infections. Her usual treatment consisted of metformin and spironolactone. The patient's blood pressure, pulse rate, and temperature were within a normal range. Physical examination of the thorax revealed no abnormalities. The abdomen was soft and nontender, with normal bowel sounds. Neurological examination showed an objective sensory deficit in the right calf, weakness 3/5 in both legs with substantial difficulty in elevating both the calf and the thigh above bed level, absent ankle jerk reflex, slight sensory deficit in perineal region, and loss of the anal tone.

Blood tests showed leucocytosis of 27,250 wbc/nL (normal range 4,5−11/nL) with a neutrophil count of 25,060/nL (normal range 2,02–7,46).

**Figure 1:**
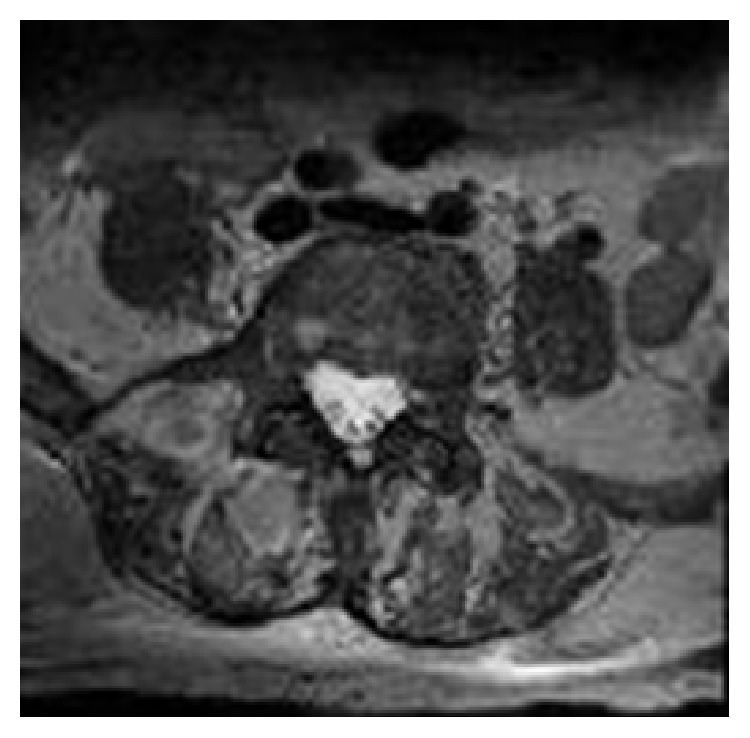
MRI: axial thoracolumbar spine T2.

**Figure 2:**
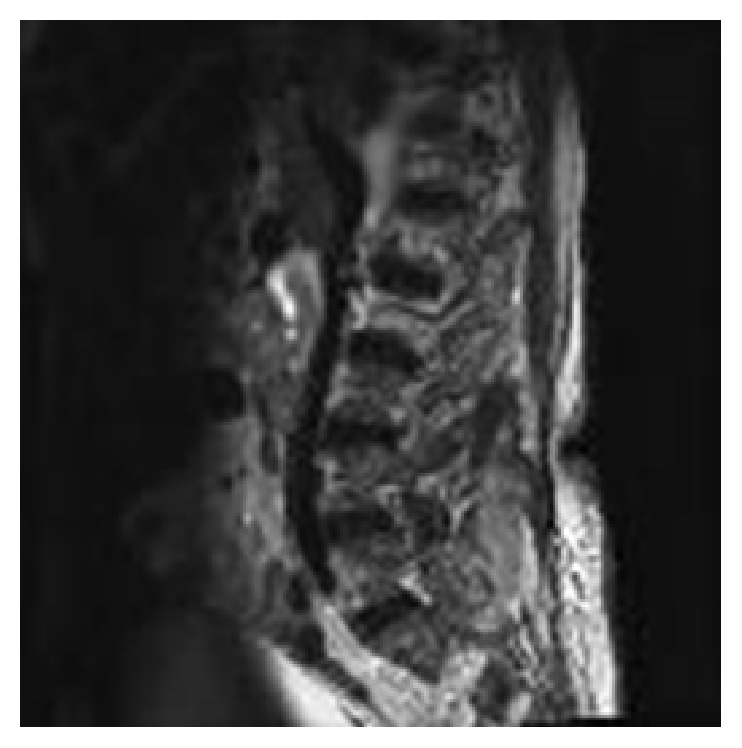
MRI: sagittal thoracolumbar spine T2.

The patient underwent an emergency magnetic resonance image (MRI) of the lower thoracic and lumbar spine without contrast based on the hypothesis of a CES. Findings were a posterior arthritis of processes L5-S1 on the right with development of an intramedullary abscess infiltrating an important part of the sacral canal towards the right iliac muscle through the L5-S1 foramen associated with compression of the nerve roots as well as a posterior extra medullary abscess of 43 × 40 mm fistulising to the right gluteal fossa with multiple intramuscular microabscesses (Figures 1 and 2).

Multiple blood cultures were drawn and a dose of oxacillin was given in the emergency department, along with glucose management. The patient was admitted to the neurosurgery ward. A decompressive laminectomy was performed the next day with extensive drainage of the abscess.

Results from the microbiology lab revealed the presence of an oxacillin sensible* Staphylococcus aureus*. Clindamycin was added to treatment by the Infectious Diseases specialists.

After a three-month follow-up, the tone of anal sphincter was reinstated. Urinary incontinence remained. Patient could walk with a walking frame, and symptoms of back pain were diminished.

## 3. Discussion and Conclusion

Only a few cases of intramedullary abscesses have been reported in literature. Those abscesses are found mostly in the lumbar region and are caused frequently by* Staphylococcus aureus* [3]. In this case report, patient had a remarkably important medical condition favouring this kind of pathology.

Symptoms of lower back pain are common in emergency rooms [4]. Persistence of the pain along with neurologically unfavourable evolution should always prompt a more detailed differential diagnosis [4]. CES is often clinically underestimated. Symptoms of a lumbar abscess are most of the time progressive, initially with fever with or without lumbar pain, followed by subtle neurological disorders of the pelvis and inferior limbs and finally heavy neurological impairment [2, 5]. MRI is the exam of choice and often diagnoses CES without yet the complete neurological entity [6–8]. Every emergency physician should be able to detect a patient presenting with a potential CES [9], as its early recognition could institute, thanks to early treatment, a more favourable outcome. In our case report, treatment was delayed because of the time of consultation. Patient waited long before presenting to our emergency department. The recovery was partial.

Our hope is for prompt recognition of lower back pain emergencies leading to immediate access to MRI and thus allowing the instauration of appropriate treatment and better outcome.
